# Usutu virus continues to spread across Europe: first report of multiple molecular detections of the USUV Africa 2 and Africa 3 lineages in free-living and captive birds in Poland, July–November 2023

**DOI:** 10.1186/s13567-025-01460-9

**Published:** 2025-02-17

**Authors:** Kamila Dziadek, Jowita Samanta Niczyporuk, Natalia Styś-Fijoł, Agnieszka Czujkowska, Krzysztof Śmietanka, Katarzyna Domańska-Blicharz

**Affiliations:** 1 Department of Poultry Diseases, National Veterinary Research Institute, Puławy, Poland; 2 Rehabilitation Centre for Birds “Bird Asylum”, Municipal Zoological Garden in Warsaw, Warsaw, Poland

**Keywords:** Usutu virus, USUV, arbovirus, vector-borne diseases, wild birds, Poland

## Abstract

The growing importance of the Usutu virus (USUV) as an emerging zoonotic viral pathogen motivated Poland to investigate the epidemiological status of USUV infections among native bird species. Consequently, out of the 357 birds tested, 34 avian individuals from different geographical areas of the country were confirmed to be USUVpositive by RT‒PCR between July and November 2023. The phylogenetic analysis revealed the first recorded occurrence of the USUV Africa 2 and Africa 3 genetic lineages in Poland.

**Supplementary Information:**

The online version contains supplementary material available at 10.1186/s13567-025-01460-9.

## Introduction, methods and results

Usutu virus (USUV) is a zoonotic mosquito-borne arbovirus of African origin whose continuous circulation has been confirmed in several European countries, including those neighbouring Poland [1–3].

Since the first documented introduction of USUV to the European continent in 1996 in Italy [4], at least 17 countries in Southern and Central Europe have reported its widespread presence, often accompanied by significant wild bird mortality, particularly during epizootics, in 2016 and 2018 [1, 2]. Tracking the evolutionary history of the USUV revealed multiple virus introductions from Africa to Europe via migratory birds, along with a continuous increase in genetic diversity among European lineages, likely due to the virus’s endemic spread across the continent [5]. While the epidemiology of USUV is being studied in many Western European countries, the current knowledge of virus occurrence in Eastern Europe remains limited. Reports of USUV cases from Central-Eastern European countries are limited to the Czech Republic, Slovakia, and Hungary [1–3], with no scientific studies available from the easternmost regions of Europe, such as Ukraine and Belarus. Prior to the first report on the detection of USUV RNA in a single mosquito pool around Poznań (Greater Poland) in April 2022 [6], the occurrence of the virus in our country could only be assumed on the basis of the results of a few serosurveys conducted several years ago [7, 8].

Therefore, to gain better insight into the current epidemiological situation of the USUV in Poland, molecular testing was carried out on free-living and captive birds showing clinical signs of a disease premortem or already found dead throughout the country.

### Research aim and sample collection

**Figure 1:**
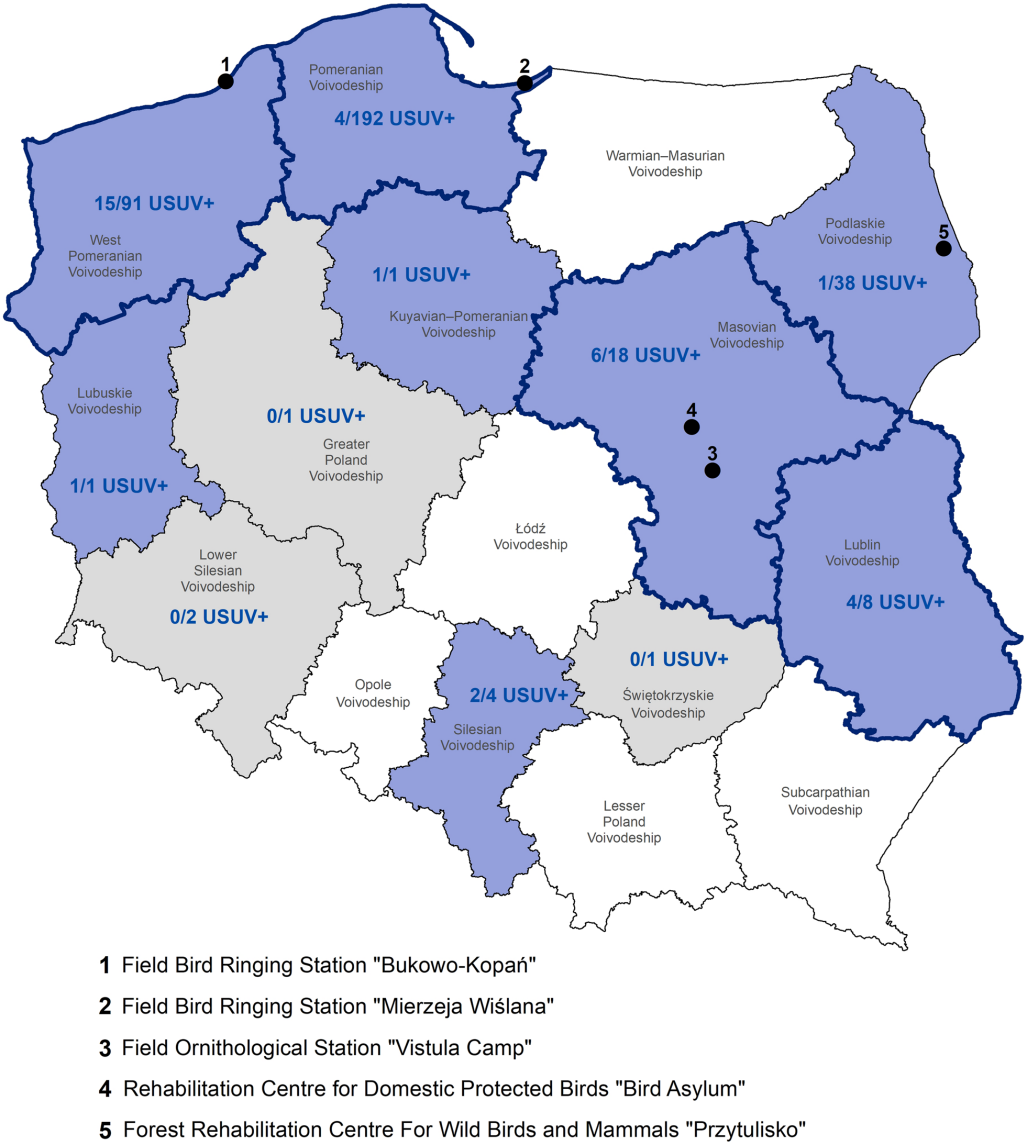
**Results of the present study on the occurrence of USUV in Poland from July–November 2023.** Graphical representation of the Polish voivodeships with the confirmed presence (blue) or absence (grey) of USUV-positive samples, along with the location of the primary ornithological facilities submitting the bird samples for this study (black dots). For the eleven surveyed voivodeships (*n* = 11), the number of USUV-positive samples compared with the total number of tested samples originating from the corresponding administrative district is provided (N_USUV+_/N_Total_). The cocirculation of the USUV Africa 2 and Africa 3 lineages is marked as bold boundaries of the respective voivodeships.

This study aimed to assess the potential occurrence of USUV in avifauna captured in Poland. For this purpose, a total of 357 dead wild and captive birds belonging to different taxonomic orders (*n* = 11) and encompassing various species (*n* = 48) were examined (Additional file 1). Avian carcasses were collected from late May to early November 2023 by experienced ornithologists working at various field ornithological/bird ringing stations or rehabilitation centres for birds located in four different voivodeships in Poland (Table 1, Figure 1). To expand the geographical area investigated, highly pathogenic avian influenza virus (HPAIV)-negative samples submitted to the National Reference Laboratory for Avian Influenza (NVRI, Puławy) in the summer and autumn seasons in 2023 from various locations in Poland were also included in the study (HPAI passive surveillance, *n* = 22). Detailed sample information is provided in Additional file 1.

**Table 1:** **Background information on avian carcasses obtained for the present study from different locations in Poland**

Sample source	Sample collection		No. of samples
	Time period	Location	
Field Bird Ringing Station “Bukowo-Kopań”	Early Sep to late Oct	West Pomeranian Voivodeship	91
Field Bird Ringing Station “Mierzeja Wiślana”	Mid-Sep to Early Nov	Pomeranian Voivodeship	192
Field Ornithological Station “Vistula Camp”	Late Jul to late Oct	Masovian Voivodeship	8
	Late Nov	Kuyavian-Pomeranian Voivodeship	1
Rehabilitation Centre for Birds “Bird Asylum”	Early Oct	Masovian Voivodeship	6
Forest Rehabilitation Centre For Wild Birds and Mammals “Przytulisko”	Late May to late Oct	Podlaskie Voivodeship	35
HPAI passive surveillance in wild birds	Late Jul	Lublin Voivodeship	6
	Late Jul	Podlaskie Voivodeship	4
	Mid-Oct	Lubuskie Voivodeship	1
	Early Jul to late Sep	Lower Silesian Voivodeship	2
	Late Jul	Silesian Voivodeship	4
	Early Jul	Świętokrzyskie Voivodeship	1
	Late Jun to mid-Sep	Masovian Voivodeship	3
	Early Jul	Wielkopolskie Voivodeship	1
Other (accidental findings)	Early Oct	Lublin Voivodeship	2
Total number of avian carcasses			357

### Laboratory examinations

Considering USUV neurotropism, priority was given to the examination of the brains, or secondarily if not available, pooled internal organs (liver, heart muscle, and spleen) of birds as individual samples. In the case of HPAIV-negative samples, the brain, internal organs, and intestines were sporadically pooled together according to the standard diagnostic procedure used for avian influenza diagnosis (Additional file 1). From the sample matrices, 20% homogenates were prepared in phosphate-buffered saline (PBS) and subsequently used for automated RNA isolation using an IndiMag Pathogen Kit (Indical Biosciences) according to the manufacturer’s instructions. To detect USUV RNA, RT‒PCR was run using USUV-specific primers targeting the partial nonstructural protein 5 (NS5) gene as described by Hubalek et al. [9]. A USUV-positive control kindly provided by the IRTA-CReSA (Barcelona, Spain) was included in the applied RT‒PCR protocol. Agarose gel electrophoresis visualization of the RT‒PCR products revealed positive results for thirty-four birds belonging to four taxonomic orders, with the highest number of USUV-positive passerine birds (*n* = 25) (Table 2).

**Table 2:** Summary of USUV-positive samples originating from free-living or captive birds (*), including spatiotemporal data

Host		Sample collection		USUV + (n)
Order	Species	Date	Location	
Passeriformes	White wagtail	12 Aug	Masovian Voivodeship	1
	Eurasian blackcap	17 Aug	Masovian Voivodeship	1
		10 Sep	West Pomeranian Voivodeship	1
	Song thrush	27 Sep	West Pomeranian Voivodeship	1
	Eurasian chaffinch	28 Sep	Pomeranian Voivodeship	1
	Common blackbird	27 Sep 22–26 Oct	West Pomeranian Voivodeship	3
		6 Oct	Lublin Voivodeship	2
	Eurasian wren	6 Oct	West Pomeranian Voivodeship	1
	Hooded crow	early Oct	Masovian Voivodeship	1
	Goldcrest	17–31 Oct	West Pomeranian Voivodeship	9
		5 Nov	Pomeranian Voivodeship	1
	Common redpoll	8–9 Nov	Pomeranian Voivodeship	2
	Eurasian jay	22 Nov	Kuyavian-Pomeranian Voivodeship	1
Anseriformes	Mallard	19 Jul	Silesian Voivodeship	2
		24 Jul	Lublin Voivodeship	2
	Mute swan	25 Jul	Podlaskie Voivodeship	1
	Bar-headed goose*	early Oct	Masovian Voivodeship	2
Charadriiformes	Common sandpiper	15 Aug	Masovian Voivodeship	1
Falconiformes	Eurasian hobby	12 Oct	Lubuskie Voivodeship	1
Total number of USUV-positive bird samples				34

### Sequencing and phylogenetic analysis

The USUV-positive RT‒PCR amplicons (495 bp) were subjected to bidirectional Sanger sequencing on a 3500 Genetic Analyser (Applied Biosystems) using the BigDye Terminator v3.1 Cycle Sequencing Kit (Thermo Fisher Scientific). Chromatogram assembly was performed in SeqScape Software v2.7 (Applied Biosystems). The obtained nucleotide sequences (GenBank accession no. PQ039613–PQ039646) were confirmed to belong to the USUV through a BLAST [10] search. The maximum-likelihood trees were subsequently generated using IQ-TREE [11] after prior alignment with MAFFT v7 [12]. At this point, sequences documented in the literature as belonging to different USUV genetic lineages [1, 3] were included as reference sequences. Automatic substitution model selection was performed, identifying the Kimura two-parameter (K2P + I) model as the best-fitting model, and the tree was constructed using the ultrafast bootstrap approximation method with 1000 replicates.

**Figure 2:**
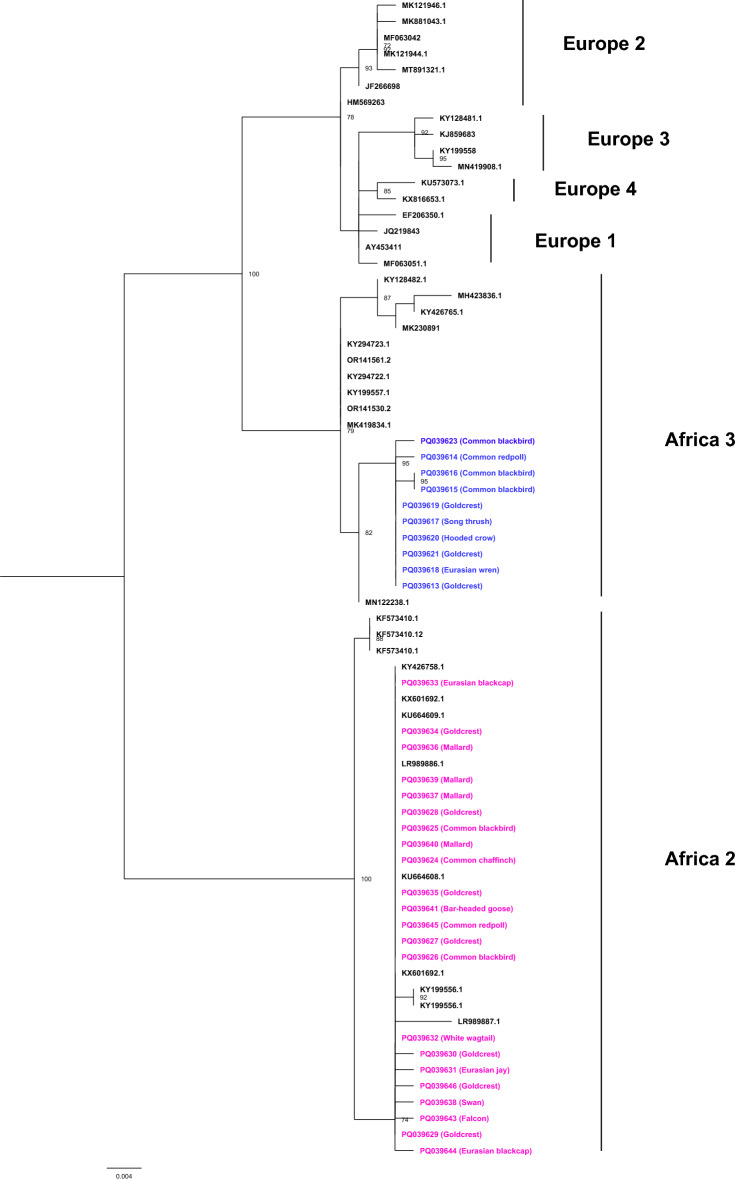
**Phylogenetic analysis of the USUV-positive samples from Poland, July–November 2023.** Maximum-likelihood tree based on the partial nucleotide sequence of the NS5 gene (471 bp), demonstrating the belonging of the Polish virus strains to the USUV Africa 2 (pink) and Africa 3 (blue) genetic lineages. IQ-TREE software [11] was used to generate the phylogenetic tree (best-fit model: K2P + I, 1000 replicates) after including nucleotide sequences with 99% similarity to the Polish viruses [10] and other lineage-specific sequences [1, 3]. Two USUV-positive samples were excluded from the phylogenetic analysis because of their shorter lengths at the 3ʹ and 5ʹ ends (PQ039622, PQ039642). Bootstrap values above 70 are shown.

The phylogenetic analysis revealed that the bird-origin viruses from Poland grouped into two distinct evolutionary clusters showing phylogenetic similarity to sequences formerly identified as USUV Africa 2 and Africa 3 genetic lineages (Figure 2). In general, the presence of USUV RNA was confirmed in samples obtained from eight out of the eleven surveyed voivodeships, with noticeable cocirculation of both virus genetic lineages in neighbouring regions in northern and central-eastern Poland (Figure 1).

## Discussion

For over twenty years, a continuous geographical expansion of various USUV genetic lineages has been observed in Europe [1–3], accompanied by an increasing number of viral infections in hosts other than birds, i.e., humans and other mammals [3, 13, 14]. Unlike other European countries, Poland does not conduct national surveillance of USUV in humans, animals, or mosquitos [13, 14]. Nevertheless, given the confirmed occurrence of USUV in neighbouring countries such as Germany, the Czech Republic, and Slovakia, along with findings from serological studies carried out within our country [7, 8], it was hypothesized that the virus probably also circulated actively across Poland.

To verify this hypothesis, carcasses of various bird species susceptible to USUV infection were acquired for molecular testing, ensuring that the widest possible geographical area of Poland was covered. As a result, the presence of the USUV was confirmed in at least half of the voivodeships spanning spatially distant regions of Poland (Figure 1), which most likely indicates the widespread occurrence of the virus across the country rather than a concentration of viral infections limited only to particular areas, e.g., near neighbouring countries with documented active USUV circulation. Furthermore, as inferred from the phylogenetic analysis, circulating virus strains belong to the African USUV genetic lineages (Africa 2 and 3) (Figure 2), whose prior emergence in other European countries, including France and Germany, was recorded several years ago [1, 2]. Thus, more advanced phylogenetic analyses based on whole genomes would help determine whether the viruses detected in Poland arose from the evolution of enzootic USUV strains circulating in Europe following cross-border spread or whether they represent a new long-distance introduction.

Since the virus is transmitted primarily by ornithophilic mosquitos from the genus *Culex* [15], multiple USUV detections at geographically distant sample collection locations in the country are likely associated with a significant presence of competent vectors for the virus in those areas. This aligns with recently reviewed data on the reported occurrence of *Culex* mosquitoes in Poland [16]. Furthermore, the latest report demonstrating the ability of female *Culex torrentium* mosquitos to act as winter reservoirs for USUV in Poland [6] clearly highlights the urgent need to establish national surveillance activities toward arbovirus vectors.

In this study, the majority of USUV-infected species were migratory birds that usually undergo seasonal long-distance journeys to nonbreeding areas southwards. The specified time points of accidental findings of the USUV-positive carcasses in Poland correspond to the fall migration period of almost all the examined bird species (August–November). Therefore, some of the reported USUV infections may have been acquired outside of Poland, perhaps in northern Europe, before the migratory route passed through Poland, which may have served only as a short-term stopover site for these birds. Considering that the susceptibility and transmission potential of northern house mosquitos to USUV has recently been confirmed in Sweden [17], it is possible that the infection sites for migrating birds in this study were regions at higher latitudes than those in Poland, where this competent vector is present. In particular, it seems to be plausible in the case of passerine birds found on the northern coast of Poland, which represents geographically the first land immediately after crossing the Baltic Sea.

Nevertheless, USUV infection in resident or partially migratory species, such as mallards and swans, has also been confirmed outside the migration season (i.e., in July) in Poland, suggesting that the virus was already present and circulated locally during the summer months.

Since most USUV-infected birds have already died and we lack information on potential disease development, i.e., the likely clinical course of the viral infection in association with any pathological findings, no conclusions can be drawn regarding differences in the pathogenicity of USUV among various bird species inhabiting Poland. The confirmation of USUV infection alone, without further information, represents a major limitation of this study, as it prevents the assessment of the impact of the virus on different hosts. Even in euthanized captive bar-headed geese showing neurological signs, the presence of USUV in their brains cannot be definitively linked as the sole cause of disease manifestation because possible coinfections with other viral pathogens or environmental factors may have worsened the clinical course. Nevertheless, severe disease development with a clear virus‒tissue lesion association due to USUV African lineages has been reported in many wild and captive bird species across Europe [18–22].

To summarize, future studies conducted in Poland should focus on serological and molecular screening of USUV-susceptible animals and arbovirus vector surveillance, with a particular emphasis on pursuing whole-genome sequencing of circulating USUV strains. Moreover, further research should investigate the relationship between USUV infection and the severity of virus-induced tissue lesions, aiming to determine the spectrum of disease susceptibility across different species as well as the evolution of the virus while it is circulating in Poland. In this manner, countrywide tracking of the spread of the virus may contribute to understanding the epidemiology of the virus worldwide.

To our knowledge, this is the first report on the multiple detections of USUV RNAs in different migratory and resident birds in Poland, specifically in terms of USUV genetic lineage annotation, i.e., Africa 2 and 3. The confirmed circulation of only African USUV genetic lineages across Poland could result from the limited number of birds tested in this study (n = 357), but it does not rule out the possible widespread presence of various European USUV lineages in the country. Taking into account the ongoing expansion of zoonotic orthoflaviviruses to the northern parts of Central Europe, as evidenced here and in a previous study from our country [23], Poland should consider implementing long-term surveillance of the USUV and West Nile virus (WNV) as an integrated approach.

## Supplementary Information

**Additional file** **1**.** Detailed sample information**. The table presents comprehensive details for all the samples used in the study, including host information and spatiotemporal data. For all USUV-positive samples, GenBank accession numbers are also provided.

## Data Availability

The datasets generated and analysed during the current study, i.e., the USUV nucleotide sequences from Poland, were deposited in the GenBank database under the following accession numbers: PQ039613-PQ039646. All sample information is included in this published article and its supplementary information file (Additional file 1).
